# Chemoradiotherapy in combination with radical surgery is associated with better outcome in cervical cancer patients

**DOI:** 10.18632/oncotarget.23165

**Published:** 2017-12-08

**Authors:** Dan Zheng, Hua-Ping Mou, Peng Diao, Xiao-Ming Li, Chuan-Li Zhang, Jing Jiang, Jia-Lian Chen, Li-Shuai Wang, Qiu Wang, Guang-Yuan Zhou, Jie Chen, Chuan Lin, Zhi-Ping Yuan

**Affiliations:** ^1^ Department of Head and Neck and Mammary Gland Oncology, and Medical Oncology, Cancer Center, West China Hospital, Sichuan University, Chengdu, Sichuan, 610041, P.R. China; ^2^ State Key Laboratory of Biotherapy/Collaborative Innovation Center of Biotherapy, West China Hospital, Sichuan University, The Cancer Center, Chengdu, Sichuan, 610041, P.R. China; ^3^ Department of Gynecology, The Second People's Hospital of Sichuan Province, Yibin City, Yibin, Sichuan, 644000, P.R. China; ^4^ Sichuan Cancer Hospital and Institute, Sichuan Cancer Center, School of Medicine, 3 University of Electronic Science and Technology of China, Chengdu, Sichuan, 610041, P.R. China; ^5^ Department of Hematology, The First Affiliated Hospital of Southwest Medical University, Luzhou, Sichuan, 646000, P.R. China; ^6^ Department of Oncology, The Second People's Hospital of Sichuan Province, Yibin City, Yibin, Sichuan, 644000, P.R. China

**Keywords:** cervical cancer, radical surgery, concurrent chemoradiotherapy (CCRT), clinical outcome

## Abstract

**Objectives:**

To retrospectively assess the influence of radical surgery following concurrent chemoradiotherapy (CCRT) on outcomes in cervical cancer (CC) patients.

**Methods:**

Patients diagnosed with cervical squamous cell carcinoma or adenocarcinoma (FIGO stages IB2 to IIB) at the Yinbin Second People's Hospital between September 2008 and September 2013, were included in this study. Patients were classified into 2 groups based on the treatment received: surgery group (CCRT plus radical surgery) and non-surgery groups (CCRT only). In addition to clinical information, inter-group differences with respect to local control rate (LCR), local recurrence rate (LRR), metastasis rate, overall survival (OS), progress free survival(PFS) and complications were assessed.

**Results:**

A total of 314 patients were included in the analysis. Parametrial invasion, pelvic lymph node metastasis, tumor diameter > 4 cm and presence of residual disease were risk factors for recurrence in the non-surgery group. In patients with risk factors, radical surgery significantly improved their clinical outcome. The 3-year/5-year LCR in the surgery and non-surgery groups was 88.3%/87.4% and 82.3%/77.5%, respectively (*P* = 0.04). The 3-year/5-year OS rate in the two groups was 87.1%/81.7% and 72.8%/67.3%, respectively (*P* = 0.001). The 3-year/5-year LRR in the two groups were 11.7%/12.6% and 17.7%/22.5%, respectively (*P* = 0.04). The metastasis rates in the two groups were 19.9% and 24.8%, respectively (*P* = 0.09).

**Conclusions:**

Surgery following CCRT could improve overall survival and progressfree survival. Radical surgery following CCRT appears to confer significant benefits including an increase in LCRs and decrease in LRR in CC patients with risk factors.

## INTRODUCTION

Cervical cancer (CC) is the fourth most common cancer worldwide for females, and the seventh most common cancer overall [1]. Many CC patients do not have resources available for undergoing surgery at the time of diagnosis. Tumor bulk, slow shrinkage after radiotherapy or residual disease, parametrial invasion, lymph node metastases are known risk factors for CC recurrence [2–5]. Residual disease post chemo-radiotherapy refers to both residual disease as well as development of fibrosis. A fraction of patients achieve a complete response (CR) during the follow up period. Treatment modalities for residual disease vary between centers and are controversial [6, 7]. In some countries, post-radiotherapy radical hysterectomy is a common practice for stage Ib-II CC while another study reported no therapeutic impact in patients achieving CR following CCRT [8, 9]. The maximum toxicity to normal tissues is exceeded for those patients receiving maximum tolerated doses during the initial chemoradiotherapy phase and interstitial implant branchy therapy can be hampered as a result. Surgery following radiotherapy has been shown to confer a survival benefit and lower local recurrence rate in CC patients [8, 10]. The first phase of treatment alters the anatomy of the pelvis tissue around the irradiation field thereby limiting treatment options for any subsequent recurrence. Thus, it is important to follow a comprehensive treatment strategy for reducing recurrence rates in patients at high-risk for recurrence. The present study aims to compare the outcomes between selected patients treated with early radical surgery after CCRT and those treated with CCRT alone.

## RESULTS

### Baseline variables

Data pertaining to 314 eligible patients were retrospectively analyzed. No statistically significant baseline inter-group differences were observed with respect to age (median, 51 vs. 55); Eastern Cooperative Oncology Group (ECOG) scores ; pathological types ; FIGO stages; radiotherapy regimen ; tumor diameters ; pelvic lymph node status ; parametrial infiltration ; and chemotherapy cycles (Table 1). Taken together, only significant difference was observed between the two groups in parametrial infiltration,

**Table 1:** Baseline variables

Characteristics	Surgery group (*N* = 163)	Non-surgery group (*N* = 151)	*P* value
Ages			0.48^*^
Median (range)	51 (26–73)	55 (28–79)	
ECOG scores			0.89^#^
≤ 2 (*N*, %)	157, 96.3%	145, 96.0%	
> 2 (*N*, %)	6, 3.7%	6, 4.0%	
Pathological types			0.45^#^
SCC (*N*, %)	136, 83.4%	121, 80.1%	
AC (*N*, %)	27, 16.6%	30, 19.9%	
FIGO stages			0.20^#^
IB2 (*N*, %)	35, 21.6%	28, 18.5%	
IIA1 (*N*, %)	27, 16.6%	39, 25.8%	
IIA2 (*N*, %)	44, 27.0%	34, 22.5%	
IIB1 (*N*, %)	41, 25.2%	30, 19.9%	
IIB2 (*N*, %)	16, 9.8%	20, 13.2%	
Radiotherapy schemes			0.08^#^
IMRT (*N*, %)	100, 61.3%	78, 51.7%	
3D-CRT (*N*, %)	63, 38.7%	73, 48.3%	
Tumor diameters			0.14^#^
≤ 4 cm (*N*, %)	103, 68.2%	103, 68.2%	
> 4 cm (*N*, %)	60, 36.8%	48, 31.8%	
Pelvic lymph node status			0.16^#^
Positive (*N*, %)	60, 36.8%	41, 25.2%	
Negative (*N*, %)	103, 63.2%	110, 72.8%	
Parametrial invasion			0.03^#^
Yes (*N*, %)	56, 34.4%	70, 46.4%	
No (*N*, %)	107, 65.6%	81, 53.6%	
Chemotherapy			0.19^#^
4 cycles (*N*, %)	13, 8.0%	19, 12.6%	
5 cycles (*N*, %)	31, 19.0%	31, 20.5%	
6 cycles (*N*, %)	119, 73.0%	62, 66.9%	

^*^Mann-Whitney test; ^#^Fisher's exact test; IMRT, intensity-modulated radiotherapy; 3D-CRT, three-dimensional conformal radiotherapy; ECOG, Eastern Cooperative Oncology Group.

### Response and recurrence rates

**Figure 1:**
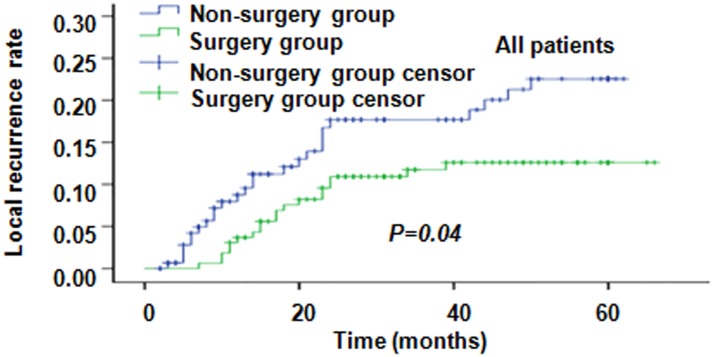
Local recurrence rates (LRR) in all the patients

**Figure 2:**
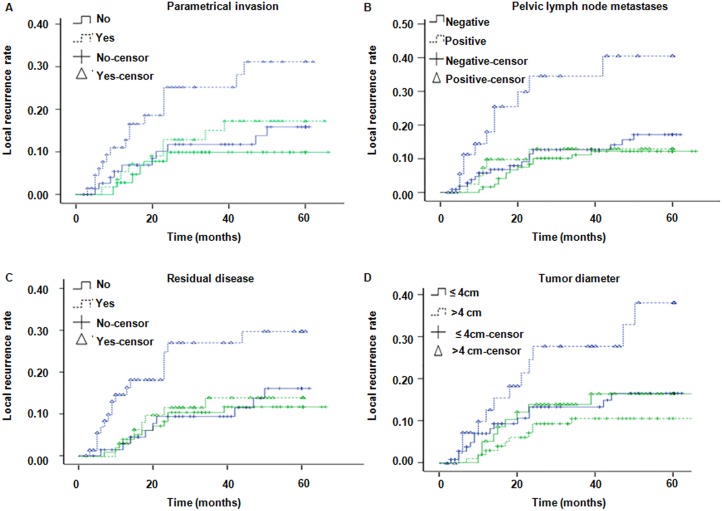
LRR in patients with or without risk factors Green line and blue line represent the surgery group and the non-surgery group, respectively. (**A**) LRR in patients with or without parametrial invasion (*P* value for surgery and non-surgery groups were 0.25 and 0.04, respectively); (**B**) LRR in patients with or without pelvic lymph node metastases (*P* value for surgery and non-surgery groups were 0.67 and 0.003, respectively); (**C**) LRR in patients with or without residual disease (*P* value for surgery and non-surgery groups were 0.71 and 0.03, respectively); (**D**) LRR in patients with or without tumor diameter > 4 cm (*P* value for surgery and non-surgery groups were 0.29 and 0.03, respectively.).

Tumor response rate after CCRT in the surgery vs non-surgery groups are shown in Supplemental Digital Content Supplementary Table 3. 3-year LCR were 88.3% and 82.3%, respectively; 5-year LCR were 87.4% and 77.5% (*P* = 0.04), respectively. At the endpoint of follow-up, the 3-year local recurrence rate (LRR) and total LRR in the surgery group were 11.7% and 12.6%, respectively, and in the non-surgery group were 17.7% and 22.5%, respectively (*P* = 0.04) (Figure 1). Furthermore, the LRR in patients with parametrial invasion (*P* = 0.04), positive pelvic lymph nodes (*P* = 0.003), residual disease (*P* = 0.03) and tumor size > 4 cm (*P* = 0.03) were significantly higher than without these factors in the non-surgery group (Figure 2 and Supplementary Digital Content Supplementary Table 1). However, there were no statistically significant differences in LRR between patients with or without risk factors, including parametrial invasion (*P* = 0.25), pelvic lymph node metastases (*P* = 0.67), residual diseases (*P* = 0.71) and tumor diameter > 4 cm (*P* = 0.29) in the surgery group (Figure 2). These results revealed that radical operation appears to significantly reduce the recurrence rate in patients with these risk factors. In the non-surgery group, 18 patients experienced LR including 11 parametrical recurrences and 7 primary recurrences. Whereas in the surgery group, 12 patients suffered LR including 6 parametrical recurrences and 6 primary recurrences. However, there was no significant difference in distant metastases rate (DMR) between the two groups. 3-year and 5-year DMR were 23.8% and 24.8% in the non-surgery group while 16.3% and 19.9% in the surgery group, respectively (*P* = 0.09). In the surgery group, 14 patients (8.59%) suffered lung metastases, 10 patients (6.13%) had supraclavicular metastases, 5 patients (3.07%) had para aortic lymph node metastases and 7 patients (4.29%) had both local/regional and distant metastases. In the non-surgery group, 16 patients (10.60%) suffered lung metastases, 8 patients (5.30%) had supraclavicular metastases, 8 patients (5.30%) had para aortic lymph node metastases and 8 patients (5.30%) had both local/regional and distant metastases.

### Outcomes of post operation histological examination

Histological examination after surgery demonstrated that 56 patients (34.4%) had parametrical invasion, 65 patients (39.9%) had residual disease, and 12 patients (7.4%) had positive margin. Whereas 13 patients whose enhanced CT or MRI show that there was residual disease but was not confirmed by histology. Furthermore, 41 patients (25.2%) showed pelvic lymph node metastases, 14 patients whose image records demonstrated positive pelvic lymph node metastases turn out to be negative in histological examination. In addition, 3.7% patients had lymphatic vessel invasion (LVSI) as determined by histological analysis.

### Overall survival (OS) and progress free survival (PFS)

**Figure 3:**
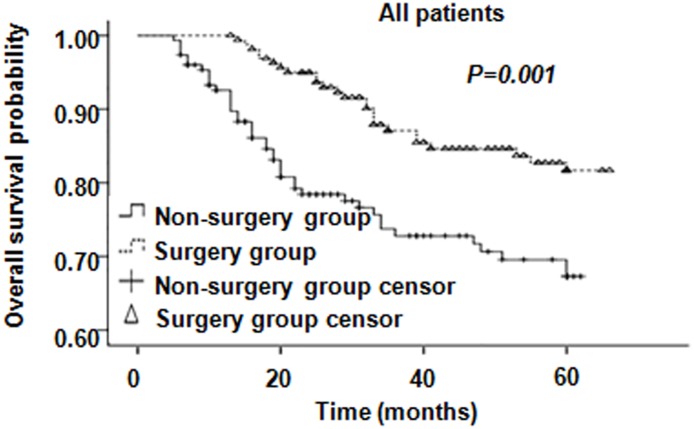
Overall survival of all patients There is no difference in OS between surgery and non-surgery groups.

**Figure 4:**
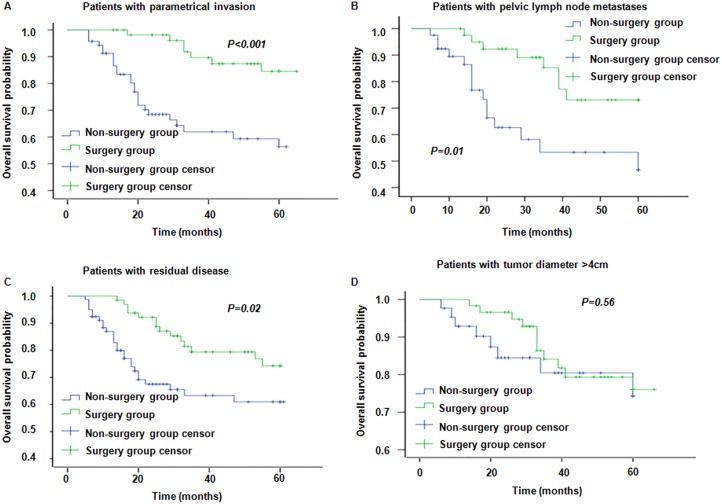
Overall survival in patients with different risk factors in surgery group and non-surgery group (**A**–**D**) represent OS for patients with parametrical invasion, pelvic lymph node metastases, residual disease and tumor diameter > 4 cm, respectively.

**Figure 5:**
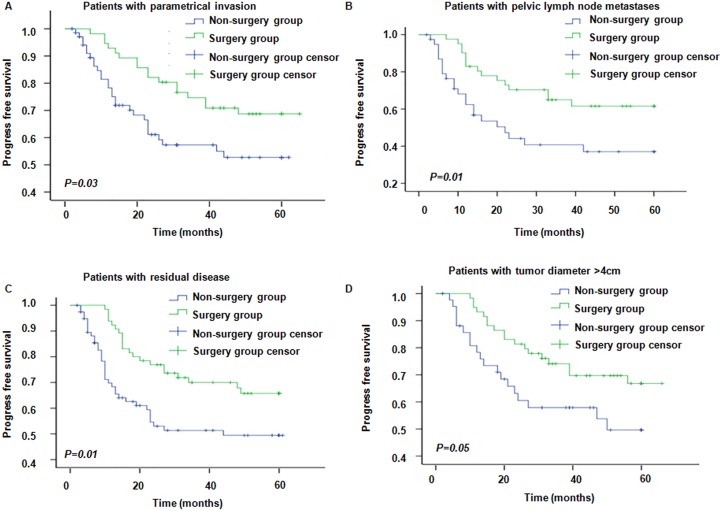
Progress free survival in patients with different risk factors in surgery and non-surgery groups (**A**–**D)** represent PFS for patients with parametrical invasion, pelvic lymph node metastases, residual disease and tumor diameter > 4 cm, respectively.

The 3-year/5-year OS rate in the surgery group was 87.1% and 81.7%, respectively; while the corresponding Figures in the non-surgery group were 72.8% and 67.3%, respectively (*P* = 0.001, Figure 3). For patients with parametrial invasion, the 3-year/5-year OS rate in the surgery group was 89.6% and 84.6%, respectively; while the corresponding Figures in the non-surgery group, were 61.9% and 56.4%, respectively (*P* < 0.0001) (Figure 4A). For patients with positive pelvic lymph nodes the 3-year/5-year OSR was 85.2% and 73.1% in the surgery group, respectively; while it was 64.7% and 60.1% in the non-surgery group, respectively (*P* = 0.09) (Figure 4B). In patients with residual disease, the 3-year/5-year OSR in the surgery group was 79.4% and 74.3%, respectively; while it was 63.3% and 61.0% (*P* = 0.02) in the non-surgery group (Figure 4C). Moreover, in patients with tumor diameter > 4 cm the 3-year/5-year OSR in the surgery group was 84.1% and 76.0%, respectively; whereas in the non-surgery groups the corresponding Figures were 80.4% and 74.3%, respectively (*P* = 0.56) (Figure 4D). These results indicate that patients with risk factors including parametrical invasion and residual disease may benefit from radical operation following chemoradiotherapy as the OS were significantly increased in the surgery group compared to the non-surgery group. However, survival benefits brought about by surgery were considerable but did not reach statistical significance in patients with pelvic lymph node metastases and bulk tumor size. The 3-year/5-year PFSR in the surgery group was 77.3% and 73.3%, respectively; while the corresponding Figures in the non-surgery group were 67.2% and 62.4%, respectively (*P* = 0.01). Moreover, PFS was significantly higher in patients with these four recurrence risk factors in the surgery group compared to the non-surgery group (Figure 5).

### Univariate and multivariate analysis

Factors including stage (RR = 1.41, *P* = 0.008), chemotherapy cycle (RR = 0.48, *P* = 0.001), parametrical invasion (RR = 2.08, *P* = 0.02) and tumor diameter > 4 cm (RR = 2.03, *P* = 0.03) were correlated with recurrence in univariate analysis by ANOVA (analysis of variance). Factors including parametrical invasion (RR = 2.03, *P* = 0.04), tumor diameter > 4 cm (RR = 2.35, *P* = 0.01), stage (RR = 1.33, *P* = 0.03) and chemotherapy cycle (RR = 0.47, *P* = 0.001) showed significant correlations with local recurrence in multivariate analysis by MANOVA (multivariate analysis of variance).

### Toxicity of CCRT and surgical complications

Hematological toxicity of grades 1, 2, 3 and 4 were documented in 289 patients, including 92 cases (29.3%), 137 cases (43.6%), 40 cases (12.7%) and 6 cases (1.9%), respectively. Febrile neutropenia was reported in 2 patients. Grade 1, 2, 3 radiation-induced enteritis and proctitis were reported in 79 cases (25.2%), 13 cases (4.1%), and 8 cases (2.6%), respectively. Furthermore, 8 (2.6%) and 7 (2.2%) patients experienced grade 1 and grade 2 radiodermatitis, respectively; and grade 1 irradiation cystitis was reported in 10 (3.18%) patients. We did not observe grade 5 toxicity in our study, and none of the patients died of CCTR toxicity. Nineteen (11.7%) patients who suffered from urinary retention recovered from this side effect within 3 weeks after radical operation with bladder functional exercises.

Collectively, these results suggest that recurrence rates may be reduced and OS and PFS increased by performing radical operation following CCRT in patients who have recurrence risk factors mentioned in this study. Patients with these risk factors had significantly lower recurrence in the surgery group as compared to that in the non-surgery group. Moreover, differences in recurrence rates can be reduced by radical operation. Among high risk patients, recurrence rates were similar in the surgery group, but they were significantly different in the non-surgery group.

## DISCUSSION

Although CCRT, the current standard of care treatment regimen for advanced CC, significantly increase OS and PFS compared to the radiotherapy alone [11], there remain risk factors leading to poor response to CCRT and increase in recurrence. There was no significant difference in the effect of CCRT between the two groups (Supplementary Table 3). Outcomes of CCRT in the two groups in the present study were comparable to other studies with similar dosing regimens of radiotherapy and chemotherapy [12–14]. All patients in our study had one or more recurrent risk factor mentioned above. The surgery group in our study had significantly lower LRR and the benefit brought about by LC partially translate into an increase in OS. A previous report observed 16.7% recurrence in patients undergoing surgery following CCRT compared to 31.7% recurrence of those that did not undergo surgery [15]. However, surgery following CCRT remains controversial because of severe complications. Coleman et al reported 38.2% severe complications in patients receiving radical hysterectomy and lymphadenectomy [16]. LR is the main reason for treatment failure that may translate to distant metastases (DM) [17]. The aim of adding surgery is not to reduce the toxicity induced by CCRT through decreasing the doses used during CCRT, but to increase the LC of patients with recurrence risk factors. In the present study, we observe a significantly higher LC that translated into a partial survival gain. However, with regard to surgery after chemoradiotherapy, interval time between CCRT and surgery and surgery type are not clear. First, a fraction of DM occurrences may not be located using modern radiology technology while detecting DM using biopsy also has limitations. Vandeperre A et al. reported 5% in PET/CT, 13% in PET and 6% in CT are false negative in para aortic lymph node metastases [18]. Therefore, we cannot absolutely exclude micro DM in the surgery group. Moreover, PD after CCRT should not be given surgery, however one patient with PD received surgery because a new pelvic lymph node metastases had been found in MRI. Second, the interval time between CCRT and surgery is controversial. Surgery after CCRT is implemented 4–8 weeks in studies including rectal cancer and CC [17, 19]. This is because fibrosis is a late adverse event occurring 3 months after CCRT that leads to difficulty in subsequent surgery [20–22]. A small number of patients received surgery within 3–4 weeks due to the patients’ requirement in our study. We found that surgery within 4 weeks following CCRT does not increase the surgery complications because edema induced by radiation is mild by this time. Third, laparoscopic hysterectomy and laparotomy were included in the present study. There were no significant differences in the effect between the two types of surgery except that complications, length of stay in hospital and bleeding volume were slightly reduced by laparoscopic. Our study did not compare the surgical type such as extrafascial hysterectomy, radical hysterectomy and extended hysterectomy. Sun L et al. reported that the most appropriate surgical approach is extrafascial hysterectomy, which had a significantly lower incidence of postoperative complication [15].

The use of surgery after CCRT is a controversial topic. To our knowledge, studies examining the effect of CCRT combined with surgery are quite limited. Houvenaeghel et al. reported a 10-year OS rate of 57.7% in these patients [23]. Another study demonstrated that CCRT combined with surgery can significantly reduce recurrence and extend survival with no severe surgical complications [24]. Our results indicate that the difference in recurrence between patients with and without risk factors can be significantly decreased by radical surgery (see Figure 2). Parametrical invasion and tumor diameter > 4 cm are important risk factors associated with prognosis (Supplemental Digital Content Supplementary Table 2). The recurrence rate was lower in the surgery group, while the risk factors contributed to LR in the non-surgery group (Figure 2). To our knowledge, this is the first study to show surgery significantly improves OS and PFS. The aim of surgery as part of a multidisciplinary treatment strategy is to achieve LC by resecting the primary lesion, and clearing or decreasing the tumor load, while chemotherapy serves to treat the circulating tumor cells (CTCs) and DM [25–30]. We find it is likely that surgery decreases local/regional recurrence rate and lymph node metastases which may contribute to the increase in OS and PFS rather than post-operation surgery increasing survival time directly as the DM rate is similar between the two groups. Tumor bulk and residual disease after radiotherapy are associated with lymph node and DM, and surgery can confer survival benefit in these patients [31, 32].

In summary, our results suggest that early and proactive radical surgery can help decrease incidence of LR and prolong OS in patients with risk factors for recurrence. Future studies with larger sample sizes are needed to confirm the clinical recommendation of radical surgery following CCRT.

## MATERIALS AND METHODS

### Patients

Patients diagnosed with cervical squamous cell carcinoma (SCC) and adenocarcinoma (AC) by cytology or histology, and classified as FIGO stage IB2-IIB with high recurrence risks assessed by gynecological examination and radiography between September 2008 and September 2013 were enrolled. Tumor diameter was measured on enhanced CT or MRI on three orthogonal plans of T2-weighted image and maximum axis was chosen. Parametrical invasion was defined as a disrupted stromal ring surrounding the uterus or parametrium irregular signal intensity in T2-weighted or diffusion-weighted image [33, 34]. Pelvic lymph node metastases were defined as high signal intensity or the shortest axis > 5 mm in abdominal pelvic CT or MRI [35]. In addition, patients who had residual disease on CT or MRI after CCRT underwent a biopsy and those with positive biopsy were scored as residual disease [36]. Residual disease was defined as definite nodes or irregular signal intensity in the primary tumor site on MRI or high signal intensity on enhanced CT scan. All patients received CCRT in Yinbin Second People's Hospital and a proportion of patients did not undergo radical operation due to economic and other complications.

Patients were categorized into surgery or non-surgery groups based on the treatment received, i.e., radical surgery following CCRT and CCRT alone, respectively. Patients underwent gynecological examination, routine blood examination, blood biochemistry, serum SCC-Ag, CEA, CA-125, liquid-based cytology or biopsy, plain chest CT, inferior abdomen and pelvic enhanced MRI at admission.

Patients with any of the following features were excluded from the study: pathological type other than SCC or AC, severe anemia (HGB < 60 g/L) pretreatment or intra-treatment, history of inferior abdominal or pelvic exenteration or irradiation, cervical stump cancer, incomplete treatment, or incomplete records.

The study was approved by the ethics committee at the Yibin Second People's Hospital. Informed consent was obtained from all patients enrolled.

### Concurrent chemoradiotherapy (CCRT)

A 6MV x-ray beam delivered by an Elekta Precise medical linear accelerator was used for external beam radiation therapy (EBRT). The radiation treatment plan consisted of 3D-CRT and IMRT. EBRT with a total pelvic dose of 46–50 Gy traditional fraction was combined with a total dose of 25–30 Gy, 5–6 Gy per fraction once a week intracavitary brachytherapy on a different day. Cisplatinum (40 mg/m^2^) was delivered once a week during the period of radiotherapy for 6 weeks. Patients were evaluated weekly with measurements of blood count, blood biochemistry, liver and renal function and clinical examination during CRT. If the white blood cell count was ≤ 2 × 10^9^/L or neutrophil cell count was 1 × 10^9^/L or platelet cell count was ≤ 50 × 10^9^/L, CCRT was stopped and recombinant human granulocyte colony-stimulating factor (rhG-CSF) and interleukin-11 were administered until the indices recovered. Vaginal douching was done daily from the beginning of radiotherapy to 1.5 years after the completion of radiotherapy to promote epidermal healing and avoid vaginal adhesions.

### Response evaluation

The tumor response was evaluated every two chemotherapy cycles according to Response Evaluation Criteria in Solid Tumors (RECIST version 1.1) until progress or recurrence or the last chemotherapy cycle.

### Radical surgery

Patients underwent a chest plant CT scan, abdominal pelvic enhancement CT or MRI or PET/CT before surgery and those PS 0–1 without severe surgery contraindication and distant metastases were treated by radical hysterectomy and pelvic lymphadenectomy within 8 weeks after CCRT completion. Biopsy was performed on patients who had scored positive for residual disease by imaging. Notable, those patients who had residual disease on image but confirmed false positive by biopsy without other recurrence risk factors were excluded. All resected tissues were sent for pathological examination.

### Follow-up

All patients had follow-up examinations every 3–6 months during the first two years, and every 6–12 months during the following 3–5 years. Physical examination, gynecological examination, routine blood test, blood biochemistry, SCC, CEA, CA-125, cervical liquid-based cytology or biopsy, pelvic enhanced MRI and plain chest CT were included in the follow-up.

### End points and statistical analysis

End points included LRR, OS, PFS and DMR. Criteria for toxicity of treatment were based on CTCAE V4.0. Statistical analyses were performed with SPSS 19.0. *P* value < 0.05 was considered to be statistically significant.

## SUPPLEMENTARY MATERIALS TABLES


