# Supplementary material for: Chemoradiotherapy in combination with radical surgery is associated with better outcome in cervical cancer patients
Source: Oncotarget. 2017 Dec 8;9(2):2866–75. doi: 10.18632/oncotarget.23165 (PMC5788687; doi:10.18632/oncotarget.23165)
Supplement: Supplementary file 1 [file oncotarget-09-2866-s001.pdf]

Chemoradiotherapy in combination with radical surgery is associated with better outcome in cervical cancer patients

SUPPLEMENTARY MATERIALS

Supplementary Table 1: LRR in patients with different risk factors

Risk factors	Surgery (%)		Non-surgery (%)		*P* value
	3-yr	5-yr	3-yr	5-yr	
Parametrial invasion	15.0	17.2	25.2	31.2	0.08
Pelvic lymph node positive	12.9	12.9	34.5	40.5	0.02
Tumor diameter > 4 cm	13.8	16.4	27.8	38.1	0.04
Residual disease	14.0	14.0	27.0	29.7	0.03

Yr, year.

Supplementary Table 2: Cox proportional hazard tests

Risk factors	Multivarite analysis		Univariate analysis	
	RR	*P* value	RR	*P* value
FIGO stage	1.33	0.03	1.41	0.008
Chemotherapy cycles	0.47	0.001	0.48	0.001
Parametrial invasion	2.03	0.04	2.08	0.02
Tumor diameter	2.35	0.01	2.03	0.03

Abbreviations: RR: risk ratio.

Supplementary Table 3: Tumor response after CCRT

Effect	Surgery group (N = 151)	Non-surgery group (N = 163)	$P^{\#}$
CR (N,%)	83, 50.9%	68, 45%	0.12
PR (N,%)	67, 41.1%	61, 40.4%	
SD (N,%)	12, 7.4%	9, 6.0%	
PD (N,%)	1, 0.6%	13, 8.6%	

[#] Mann-Whitney test.
